# An Incidental Finding of Suppurative Appendicitis During Colonoscopy

**DOI:** 10.7759/cureus.43159

**Published:** 2023-08-08

**Authors:** Ayat Idris, Khalid Al Shamousi, Ahmed Alwassief, Adil Al Zadjali, Katarzyna Laszczak

**Affiliations:** 1 Gastroenterotolgy Unit, Department of Medicine, Sultan Qaboos University Hospital, Muscat, OMN; 2 General Surgery, Sultan Qaboos University Hospital, Muscat, OMN; 3 Gastroenterotolgy, Medical University of Lublin, Lublin, POL

**Keywords:** suppurative, asymptomatic, endoscopy, appendicitis, colonoscopy

## Abstract

Colonoscopic diagnosis of acute appendicitis is extremely rare. Although a few appendicitis cases were reported in literature following colonoscopy, we present a case today of a patient who underwent elective colonoscopy for colorectal cancer screening. The presence of an inflamed appendiceal orifice with projecting pus was documented, and the patient was referred to the surgical team for intervention. Endoscopic and intra-operative results are also illustrated.

## Introduction

Acute appendicitis is one of the most common causes of acute abdomen. It occurs in 100 cases per 100,000 patients per year [1]. Classic symptoms include an acute presentation with right iliac fossa (RIF) pain, anorexia, nausea, constipation, and vomiting; however, this classic presentation only occurs in 50% of people. On the other hand, asymptomatic infection is not uncommon [2]. Colonoscopic diagnosis of asymptomatic early acute appendicitis is extremely rare. It has been suggested that appendicitis may develop as a result of colonoscopy [3]. Acute appendicitis after lower GI endoscopy has an estimated incidence of 3.8 to 4.9 per 10,000 colonoscopies [4]. Establishing causality is difficult, but there is an association between the two entities. Here we present a case of early-stage suppurative appendicitis discovered incidentally during elective screening colonoscopy.

## Case presentation

**Figure 1:**
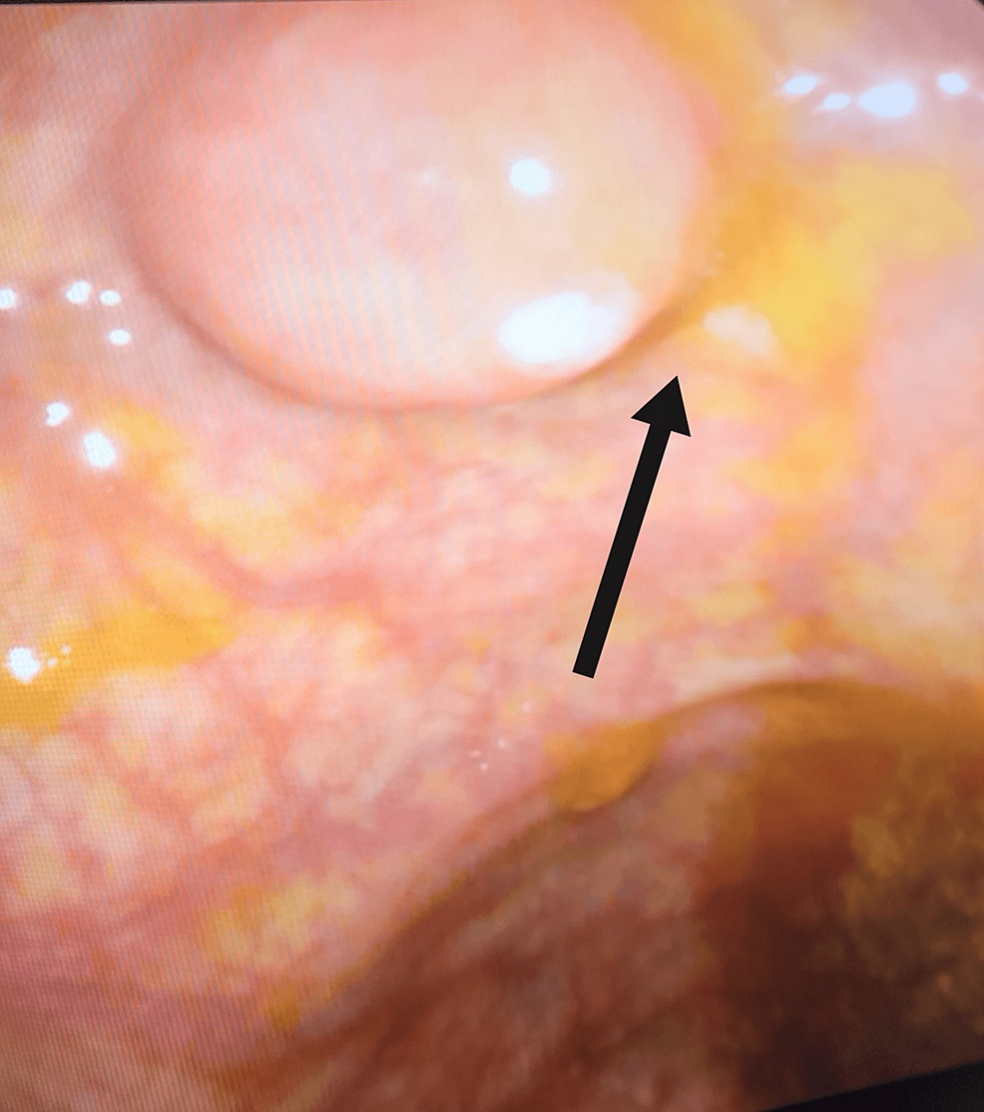
Inflamed appendiceal orifice extruding pus during colonoscopy (arrow).

**Figure 2:**
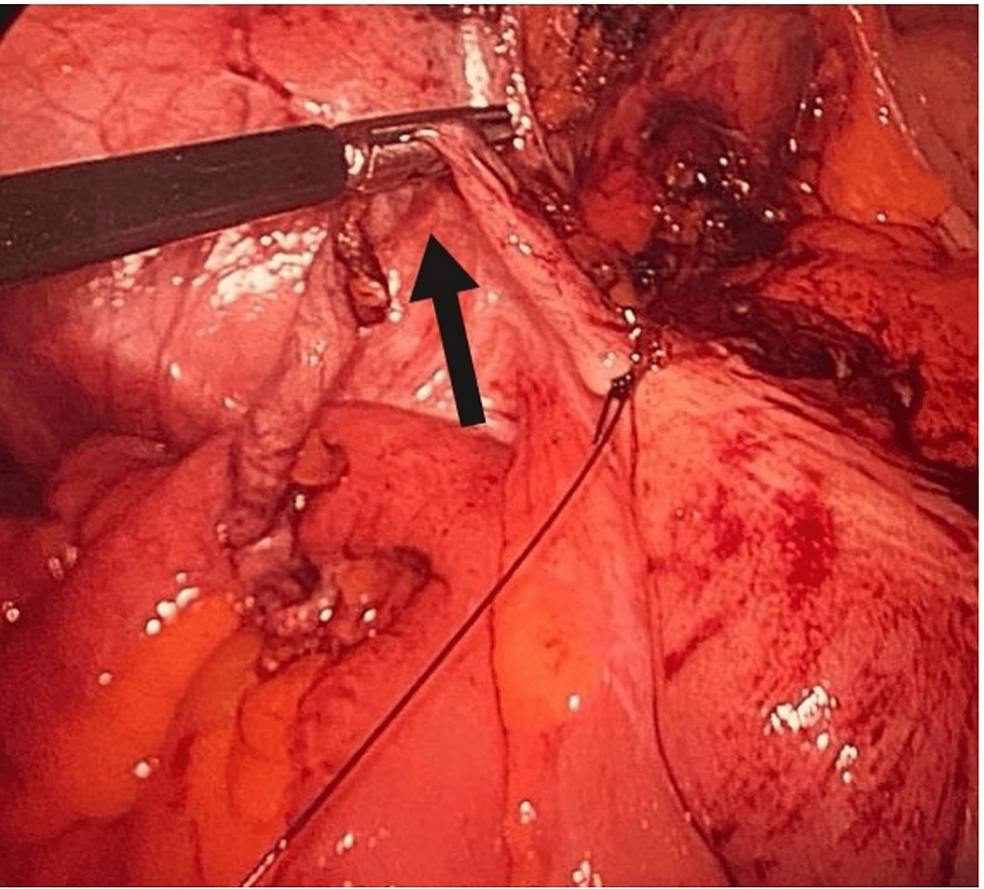
Intra-operative findings of the inflamed appendix (arrow).

A 56-year-old woman with a history of dyslipidemia, prediabetes, subclinical hypothyroidism, and multilevel disc herniation was booked for an elective diagnostic colonoscopy after a positive fecal occult blood screening. She presented to the endoscopy department with a history of mild chronic abdominal pain mainly in the epigastric area for one year. Colonoscopy revealed a protruding caecal opening that resulted in pus discharge when an attempt was made to biopsy the region (Figure 1). Drainage was not attempted endoscopically and she was referred to the general surgery team for evaluation. Physical examination showed a soft abdomen with no tenderness, guarding, or rebound tenderness over the right iliac fossa. Her full blood count and C-reactive protein levels are shown in Table 1 and computed tomography CT of the abdomen showed only a thickened appendicular wall. The patient was discharged on oral antibiotics (ciprofloxacin 500 mg every 12 hours and metronidazole 500 mg every eight hours) according to the surgical team's decision. Three days later she presented to the emergency department with acute severe abdominal pain in the right lower quadrant and fever; her blood tests are shown in Table 2. She was diagnosed with acute appendicitis and subsequently underwent a laparoscopic appendectomy. The intraoperative findings and the histopathology confirmed the diagnosis of a chronically inflamed appendix (Figure 2). She made a full recovery and was discharged a few days later.

**Table 1:** Summary of laboratory results on the day of colonoscopy.

Test	Result	Normal range
Hemoglobin (g/dL)	13.3	12.1 - 15.1
White blood cell count (10^9/L)	8.3	4.5 - 11.0
Platelets count (10^9/L)	248	150 - 300
C-Reactive protein (mg/L)	26	<6

**Table 2:** Summary of laboratory results on the day of the operation.

Test	Result	Normal range
Hemoglobin (g/dL)	13.3	12.1 - 15.1
white Cell Count (10^9/L)	4.4	4.5 - 11.0
Platelets count (10^9/L)	291	150 - 300
C-Reactive Protein (mg/L)	11	<6

## Discussion

Acute appendicitis is mainly caused by luminal obstruction, especially in elderly patients. In young people, the main cause is suspected to be lymphoid follicular hyperplasia, which can be triggered by a local infection [5-6]. The appendix may remain blocked, which then leads to perforation and abscess formation. Alternatively, the obstruction may resolve spontaneously while residual infection persists, resulting in chronic appendicitis [7]. This may explain the asymptomatic status (no serositis as pus flows to the cecum). Patients with acute appendicitis usually present with periumbilical pain radiating to the right iliac fossa, anorexia, nausea, vomiting, low-grade fever, tenderness, and alertness [8]. There are few asymptomatic cases reported in the literature [2-8]. Diagnosis is based on history, physical examination, laboratory tests, and imaging tests, including abdominal ultrasound, computed tomography, and magnetic resonance imaging. A colonoscopy is not one of the standard imaging procedures for appendicitis [9]. Our case represents a rare situation in which purulent silent inflammation was first documented during a colonoscopy. Laparoscopic appendectomy together with broad-spectrum antibiotics such as piperacillin-tazobactam in monotherapy or cephalosporins or fluoroquinolone with metronidazole are the most common treatment options [10]. In fact, there are few reports of appendicitis after colonoscopy, presumably as a result of precipitation of intestinal ischemia or microbiome alteration [11-12]. Most of the reported cases of appendicitis following colonoscopy were documented within a week or two of colonoscopy and within the context of a normal appendiceal orifice [13]. On the other hand, a retrospective study characterized the role of colonoscopy in diagnosing appendicitis: the common findings of established appendicitis at colonoscopy include edema of the appendiceal orifice, bulging, and drainage of pus through the orifice [9]. In the present case, we documented erythema, bulging, and extrusion of pus through the appendiceal opening into the cecum opening during colonoscopy. In addition, the CT abdomen showed only appendiceal thickening with no fat stranding or appendicoliths, so this case is clearly subclinical appendicitis discovered incidentally during colonoscopy and not a colonoscopy complication. Endoscopy as a treatment option has been evaluated in various studies. Although acute appendicitis is a common condition, there are few reported cases of asymptomatic acute appendicitis [2-8]. There were only three cases where colonoscopy was used as a treatment, mainly to relieve appendectomy [14-15]. Interestingly, two of the reported cases were asymptomatic. On the other hand, in delayed or atypical appendicitis, a colonoscopy is one of the best diagnostic tools [9].

## Conclusions

Although cases of appendicitis after colonoscopy have been reported, to our knowledge, we have documented purulent acute appendicitis endoscopically for the first time. Therefore, endoscopists must be aware of the potential for appendicitis during colonoscopy and its occurrence so that appropriate treatment can be promptly instituted to avoid serious complications.

## References

[1] Humes DJ, Simpson J (2006). Acute appendicitis. BMJ.

[2] Pereira X, Romero-Velez G, Dickinson G, Mandujano CC (2020). Endoscopic diagnosis of early acute appendicitis in an asymptomatic patient. ACG Case Rep J.

[3] Vender R, Larson J, Garcia J, Topazian M, Ephraim P (1995). Appendicitis as a complication of colonoscopy. Gastrointest Endosc.

[4] Al Hillan A, Mohamed M, Chien D, Alshami A, Arif F (2020). Postcolonoscopy appendicitis: a delayed complication. Cureus.

[5] Jones MW, Lopez RA, Deppen JG (2023). Appendicitis. StatPearls.

[6] Bhangu A, Søreide K, Di Saverio S, Assarsson JH, Drake FT (2015). Acute appendicitis: modern understanding of pathogenesis, diagnosis, and management. Lancet.

[7] Kothadia JP, Katz S, Ginzburg L (2015). Chronic appendicitis: uncommon cause of chronic abdominal pain. Therap Adv Gastroenterol.

[8] Petro M, Minocha A (2005). Asymptomatic early acute appendicitis initiated and diagnosed during colonoscopy: a case report. World J Gastroenterol.

[9] Chang HS, Yang SK, Myung SJ (2002). The role of colonoscopy in the diagnosis of appendicitis in patients with atypical presentations. Gastrointest Endosc.

[10] Shang Q, Geng Q, Zhang X, Guo C (2017). The efficacy of combined therapy with metronidazole and broad-spectrum antibiotics on postoperative outcomes for pediatric patients with perforated appendicitis. Medicine (Baltimore).

[11] Shaw D, Gallardo G, Basson MD (2013). Post-colonoscopy appendicitis: A case report and systematic review. World J Gastrointest Surg.

[12] Drago L, Toscano M, De Grandi R, Casini V, Pace F (2016). Persisting changes of intestinal microbiota after bowel lavage and colonoscopy. Eur J Gastroenterol Hepatol.

[13] Chae HS, Jeon SY, Nam WS, Kim HK, Kim JS, Kim JS, An CH (2007). Acute appendicitis caused by colonoscopy. Korean J Intern Med.

[14] Benatta MA (2012). Incidental Diagnostic and Treatment of a Suppurative Appendicitis at Colonoscopy. Case Rep Med.

[15] Liu CH, Tsai FC, Hsu SJ, Yang PM (2006). Successful colonoscopic drainage of appendiceal pus in acute appendicitis. Gastrointest Endosc.

